# Are Global and Regional Improvements in Life Expectancy and in Child, Adult and Senior Survival Slowing?

**DOI:** 10.1371/journal.pone.0124479

**Published:** 2015-05-18

**Authors:** Ryan J. Hum, Stéphane Verguet, Yu-Ling Cheng, Anita M. McGahan, Prabhat Jha

**Affiliations:** 1 Center for Global Engineering and Department of Chemical Engineering and Applied Chemistry, University of Toronto, Toronto, Ontario, Canada; 2 Department of Global Health and Population, Harvard T.H. Chan School of Public Health, Boston, Massachusetts, United States of America; 3 Rotman School of Management and Munk School of Global Affairs, University of Toronto, Toronto, Ontario, Canada; 4 Center for Global Health Research, St Michael’s Hospital, University of Toronto, Toronto, Ontario, Canada; Johns Hopkins University, UNITED STATES

## Abstract

Improvements in life expectancy have been considerable over the past hundred years. Forecasters have taken to applying historical trends under an assumption of continuing improvements in life expectancy in the future. A linear mixed effects model was used to estimate the trends in global and regional rates of improvements in life expectancy, child, adult, and senior survival, in 166 countries between 1950 and 2010. Global improvements in life expectancy, including both child and adult survival rates, decelerated significantly over the study period. Overall life expectancy gains were estimated to have declined from 5.9 to 4.0 months per year for a mean deceleration of -0.07 months/year^2^; annual child survival gains declined from 4.4 to 1.6 deaths averted per 1000 for a mean deceleration of -0.06 deaths/1000/year^2^; adult survival gains were estimated to decline from 4.8 to 3.7 deaths averted per 1000 per year for a mean deceleration of -0.08 deaths/1000/year^2^. Senior survival gains however increased from 2.4 to 4.2 deaths averted per 1000 per year for an acceleration of 0.03 deaths/1000/year^2^. Regional variation in the four measures was substantial. The rates of global improvements in life expectancy, child survival, and adult survival have declined since 1950 despite an increase in the rate of improvements among seniors. We postulate that low-cost innovation, related to the last half-century progress in health–primarily devoted to children and middle age, is reaping diminishing returns on its investments. Trends are uneven across regions and measures, which may be due in part to the state of epidemiological transition between countries and regions and disparities in the diffusion of innovation, accessible only in high-income countries where life expectancy is already highest.

## Introduction

Life expectancy is an overall measure of population health. The approximate doubling of life expectancy over the last century is a demonstration of substantial scientific and public health progress [1,2]. Measuring the pace at which health indicators increase is important to anticipate increases in longevity, to project rising costs of health care in our modern societies, and to mark progress in achieving objectives such as the reductions in child mortality stipulated in the Millennium Development Goals (MDGs).

In an influential paper, Oeppen and Vaupel [3] demonstrated that female life expectancy in the best performing countries in the world rose consistently and steadily at about three months per year from 1840 to 2000. This finding was interpreted to contest the argument by Fries [4] and Olshansky et al. [5] that improvements in life expectancy would be capped by biological limits. The prospect of unlimited increases in life expectancy was further supported by White [6], which reported a similarly consistent and steady rate of improvements in life expectancy among both men and women in 21 industrial countries between 1955 and 1995; White’s research was further supported by Lee [7]. Subsequently, Vallin and Meslé [1] pointed out that this consistent and steady pace of improvement could be parsed into a series of sequential segments, each of which reflected distinct and important health transitions. Specifically, from the late nineteenth century to the 1960s, medical discoveries stemming from the work of Louis Pasteur such as antiseptics, vaccines and antibiotics decreased infectious diseases in the high-income countries–predominantly reducing child mortality [1,2]. Most recently, life expectancy gains in high-income countries have accrued from declines in adult mortality via decreases in premature cardiovascular mortality as a result of the reduction in tobacco consumption, improvements in diagnosis, and the administration of pharmacological therapies [8–10]. Vallin and Meslé [1] ultimately concluded that an unending prospective, continual rise in maximum life expectancy (particularly at a pace of three months per year) may not be achievable. Notwithstanding the historical steady rise in life expectancy, the principal argument refuting a limit to longevity is the absence of ‘leveling’ off (or diminishing improvements) of life expectancy gains [3,11,12].

Most global public health research on summary measures of health such as life expectancy has focused heavily on the numerical values of these measures (i.e. prevailing years) rather than their trends or rates of improvements (i.e. months gained per year in average life expectancy in a population born in the year, or alive and at a particular age in the year), although a literature has begun to emerge that focuses on trends [13–20], and particularly to describe progress toward achieving goals such as the MDGs [21,22]. Demographic analyses [23–25] commonly consider trends as a marker of the efficacy of particular policy interventions designed to improve health over short periods of time. When rates of improvements of health outcomes are responsive to changes in the determinants of health, they offer a valuable dependent variable for analysts seeking to understand the influence of the determinants of health; this is the premise of studies using first differences or difference-in-differences analysis [26].

Recently, Bloom and Canning [27] studied the relationship between the life expectancy improvements from 1963 to 2003. When mortality due to AIDS was removed, they found that countries with higher 1963 life expectancy improved more slowly, whereas countries with lower 1963 life expectancy improved at rates indifferent to their initial life expectancy levels. This paper suggests that a country that starts with a high level of health is likely to persist with a high level of health but with diminishing marginal gains. This idea of slower changes in life expectancy for countries already achieving high life expectancy is formally operationalized by the United Nations (UN) Population Division, whose projections assume higher annual gains in life expectancy for countries in less developed regions compared with those in more developed regions [28,29].

**Fig 1:**
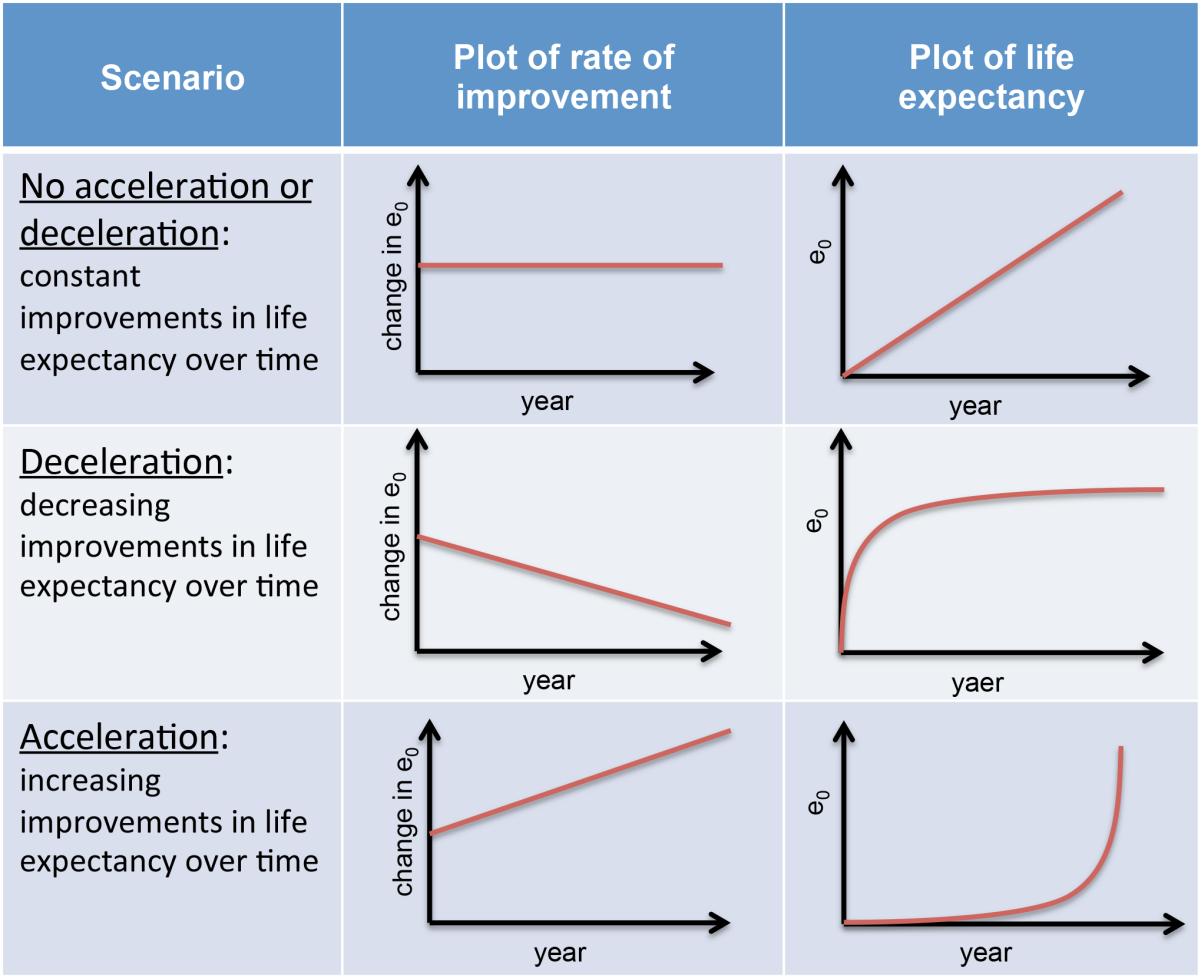
Terminology and graphical representation of different scenarios of growth in life expectancy.

In this paper, we consider the evolution of the absolute rates of improvements in life expectancy, child survival, adult survival, and senior survival for 166 countries over the period 1950–2010. We use a standard calculus approach whereby: a constant rate of improvement (e.g. neither acceleration nor deceleration) leads to a steady linear increase of life expectancy over time (Fig 1, top); a positive rate of improvement leads to an ‘acceleration’ scenario corresponding to increasing marginal returns of life expectancy over time (Fig 1, bottom); a negative rate of improvement leads to a ‘deceleration’ scenario corresponding to diminishing marginal returns of life expectancy over time (Fig 1, middle). By using explicitly rates of improvements, we quantify global diminishing improvements in three health measures—life expectancy, child survival and adult survival—but accelerating improvements in seniors’ survival (ages 60–80). We point to the notion that diminishing improvements in global life expectancy are not solely accompanied with diminishing returns in reducing child mortality by quantifying global diminishing improvements for both child and adult survival. Notwithstanding the declines in child and adult survival improvements, gains in older age continue to improve but with expectedly less impact on changes in life expectancy.

## Methods

We examine life expectancy at birth (e_0_), child survival (probability of surviving from birth to age 5, i.e. 5p0), adult survival (probability of surviving from age 15 to age 60, i.e. 45p15) and senior survival (probability of surviving from age 60 to 80, i.e. 20p60)—for both males and females combined. For consistency, we use the 2012 revision of the World Population Prospects (WPP2012) for all available mortality estimates (sex-combined for the twelve five-year intervals from 1950 to 2010) [29]. For seniors, we utilize the abridged life tables to directly calculate 20p60 [29]. 166 countries were used in the analysis, representing all available countries in the WPP2012, with populations greater than 500,000 in the year 2005 [29]. The list of all countries included in the analysis is available in S1 Text. Countries were organized into 6 regions (Asia, Eastern Europe & Central Asia, High-income, Latin America & the Caribbean, Middle East, and sub-Saharan Africa). A list of all six regions included in the analysis is available in S1 Text. A sensitivity analysis was also completed with the 113 countries greater than 5 million with no major change in the conclusions.

### Rate of life expectancy improvement

The public UN database releases life expectancy averages for each of the twelve five-year intervals *Y* from 1950–54, 1955–59, 1960–64, 1965–69, 1970–74, 1975–79, 1980–84, 1985–89, 1990–94, 1995–99, 2000–04, and 2005–09. The choice of the starting year was dictated by the data availability in the UN database. For country *i* in time interval *Y*, we computed a county’s average annual improvement, ν_*i*,*Y*_, of e_0_ from the first derivative of the levels of e_0_ (*e*_*0*,*i*,*Y*_):

$$\nu_{i,Y}=\frac{de_{0,i,Y}}{dY}\approx \frac{e_{0,i,Y+1}-e_{0,i,Y}}{Y_{t+1}-Y_{t}}\approx \frac{e_{0,i,Y+1}-e_{0,i,Y}}{5}$$ (1)

### Linear mixed effect model

A linear mixed effect model [30] was developed to investigate the relationship between improvement of e_0_ and time between 1950–2010. We start with the basic model:

$$v_{i,Y}=\gamma_{0}+\gamma_{1}t+\varepsilon_{1,i,Y}$$ (2)

where *v*_*i*,*y*_ is the country’s average annual improvement (converted to months per year), *t* is time (in unit years, where the five-year period 1950–1954 is *t* = 0, the period 1955–1959 is *t* = 5…), *y*_1_ is the rate of change of improvement (in units months per year^2^; the unit “months per year^2^” (i.e. months per year per year or months per year*year) is an acceleration unit. An analogy in driving is the acceleration of a car in meters per second*second (or second^2^)) and *ε*_1,*i*,y_ is an error term. The time term (rather than year) was used in the model so that the intercept (*γ*_0_)could be used to determine the rate of improvement at the beginning of the study period (in units months per year). Across all regions (*R*), the coefficients *γ* have a normal distribution with a given mean and variance. At the regional level, the variation in the intercept and slope is expressed as:

$$\gamma_{0}=\mu_{0}+\delta_{0,R}$$ (3)

$$\gamma_{1}=\mu_{1}+\delta_{1,R}$$ (4)

The terms *μ* express the fixed (global) average across all countries and *δ*_*R*_ is the deviation from the global average for a given region *R* (region effect), assumed to be normally distributed with mean zero and *cov*(*δ*_0,*R*_;*ε*_0,*i*,*y*_) = *cov*(*δ*_1,*R*_;*ε*_1,*i*,*y*_) = 0. This model was further developed to incorporate a third-level country (*i*) specific random effect (*τ*), such that:

$$\delta_{0,R}=\beta_{0}+\tau_{0,i}$$ (5)

$$\delta_{1,R}=\beta_{1}+\tau_{1,i}$$ (6)

Using Eqs (4) and (5) and the derived regression estimates, a Monte Carlo simulation (with 1000 iterations) was run to determine confidence intervals for each specific regional rate of change of life expectancy improvements [31]. In this model, a ‘decelerating’ global life expectancy scenario would be represented as a negative, statistically significant *γ*_*1*_ term; whereas a not statistically significant *γ*_*1*_ term would represent a steady growth (e.g. no acceleration or deceleration) scenario.

To validate the stability of the model, we repeated the analysis for the period 1960–2010 and for male and female (separated) life expectancy. To validate the predictability of the model (for future projections beyond 2010), we repeated the analysis by developing a model for the period between 1950–2004 and evaluated the model predictions for the following subset of years 2005–2009. To test the non-linearity in model (2) above and determine relative model fitness [32], regressions were repeated with two quadratic variants of Eq (2) of the form:

$$v_{i,Y}=\gamma_{0}+\gamma_{1}t^{2}+\varepsilon_{1,i,Y}$$ (7)

$$v_{i,Y}=\gamma_{0}+\gamma_{1}t^{2}+\gamma_{1}t+\varepsilon_{1,i,Y}$$ (8)

By using Eq (1), we continue the tradition of Oeppen and Vaupel [3], Vallin and Meslé [1], White [6], and Lee [7] and calculate the absolute change in life expectancy per year. Recognizing that rates of improvements can be calculated as absolute or relative changes, we repeated the analysis using relative improvements, such that Eq (1) is modified to become an annual percentage change:

$$\nu_{i,Y}=100*\frac{de_{0,i,Y}}{e_{0,i,Y}*dY}\approx 100*\frac{e_{0,i,Y+1}-e_{0,i,Y}}{e_{0,i,Y}*\left(Y_{t+1}-Y_{t}\right)}$$ (9)

We repeated the same analysis for 5p0 and 45p15 and 20p60. All analyses were conducted with the R statistical software (www.r-project.org) and SAS. A restricted maximum likelihood approach was used to estimate the equations.

## Results

### Rate of life expectancy improvement

**Fig 2:**
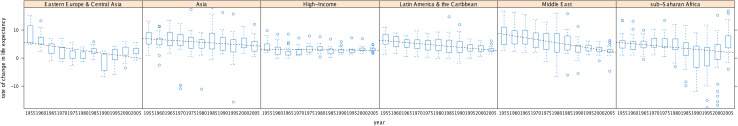
Regional change in life expectancy (months per year), 1950–2010. Note: mean regional trends in changes in life expectancy are indicated by the black dashed lines.

The results of the linear mixed effects model for the changes in life expectancy improvements for the period 1950–2010 are summarized in Table 1. A statistically significant ‘time’ term represents a non-linear relationship between level of life expectancy and time; a negative value demonstrates diminishing rate of improvements in life expectancy over time (Fig 1, bottom). The global mean improvement *γ*_1_ is decreasing from 1950–54 to 2005–09, from about 5.9 months per year to 4.0 months per year. The global mean rate of improvement is -0.070 months/year^2^ (95% CI: -0.10, -0.04, p<0.001) with an (hypothetical) extrapolated net zero growth by the year 2055. Expectedly, this is consistent with a simple linear model where diminishing improvements were estimated at -0.070 months/year^2^ (-0.08; -0.06). While all regional estimates show overall diminishing improvements (Fig 2), the region with the smallest diminishing improvements (and not statistically significant) is High-income; it was also the region with the lowest rate of improvement in 1950–54 (Table 2). The Middle East region had the highest diminishing improvements, although the variance was high among the countries within this region (Fig 2). S1 Fig shows the evolution of improvements in life expectancy from 1950 to 2010 for all countries. The variance in country effects *τ*_0,_ *c* and *τ*_1,_ *c* were -0.054 months per year (p<0.10) and 0.003 months/year^2^ (p<0.05) respectively.

**Table 1:** Results of the linear mixed effect model for the relationship between rate of improvements in life expectancy, child, adult and senior survival, and time, for 1950–2010.

	Life Expectancy	Child Survival	Adult Survival	SeniorSurvival
Marginal R^2^	0.05	0.07	0.04	0.03
Conditional R^2^	0.18	0.33	0.10	0.08
Intercept (Rate of improvement at beginning of study period)	5.9*** (0.72)	4.4*** (0.66)	4.8*** (0.69)	2.4*** (0.24)
Time (Trend in rate of improvement)	-0.070*** (0.016)	-0.063*** (0.009)	-0.075*** (0.016)	0.031‘ (0.021)
Mean rate of improvement at end of study period	4.0 (0.26)	1.6 (0.14)	3.7 (0.40)	4.2 (0.28)

‘ indicates p ~ 0.15,

* indicates p < 0.05,

** indicates p < 0.01;

*** indicates p < 0.001.S.E. (standard error) is in parentheses.

The intercept is in units of months per year or deaths per 1000 per year. The time effects are in units of months per year^2^ or deaths per 1000 per year^2^.

**Table 2:** Mean rate of improvement in 1950–54 and the mean regional trends in improvements in life expectancy, child survival, adult and senior survival from 1950–2010.

	Life Expectancy	Child Survival	Adult Survival	Senior Survival
Rate of improvement in 1950 to 1954				
Asia	6.19* (1.04)	5.27* (0.94)	6.38* (1.07)	2.03* (0.81)
Eastern Europe & Central Asia	5.19* (0.72)	3.99* (0.68)	3.05* (0.68)	2.41* (0.23)
High-income	3.54* (0.73)	1.99* (0.68)	3.00* (0.68)	2.48* (0.24)
Latin America & the Caribbean	6.18* (0.74)	4.19* (0.64)	4.99* (0.69)	3.08* (0.24)
Middle East	8.11* (0.71)	6.71* (0.65)	6.01* (0.69)	2.57* (0.24)
sub-Saharan Africa	5.47* (0.72)	4.24* (0.69)	5.23* (0.67)	2.03* (0.24)
Regional trends in improvement				
Asia	-0.059* (0.016)	-0.067* (0.009)	-0.070* (0.015)	0.049* (0.021)
Eastern Europe & Central Asia	-0.092* (0.015)	-0.076* (0.009)	-0.066* (0.016)	-0.015 (0.021)
High-income	-0.022 (0.025)	-0.034‘ (0.018)	-0.040* (0.016)	0.1122* (0.022)
Latin America & the Caribbean	-0.068* (0.016)	-0.062* (0.009)	-0.076* (0.016)	0.039‘ (0.021)
Middle East	-0.108 (0.759)	-0.103 (0.681)	-0.083* (0.016)	0.030 (0.021)
sub-Saharan Africa	-0.067* (0.026)	-0.034‘ (0.018)	-0.110* (0.028)	-0.008 (0.031)

‘ indicates p ~ 0.10,

* indicates p < 0.05. S.E. (standard error) is in parentheses.

The rate of improvement in 1950–54 is in units of months per year or deaths per 1000 per year. The regional trends in improvement are in units of months per year^2^ or deaths per 1000 per year^2^.

For most regions, the rate of improvements in life expectancy appears to diminish somewhat linearly with time with the exception of sub-Saharan Africa, which shows a substantial decline from 1980–84 through 1995–99, followed by a sharp increase through 2005–09 (Fig 2). This pattern is evident in countries with high HIV prevalence (those with greater than 5% in any given year during the study period [33]; S2 Fig). Similar to sub-Saharan African countries, many former Soviet state countries also exhibited a mortality shock starting in the 1980s. While the magnitude of this mortality shock varied between countries, the post-shock average rate of improvements returned to approximately the pre-shock values.

Other specific countries that exhibited pronounced shock periods include: Bangladesh, Bosnia and Herzegovina, Cambodia, China, Iran, Iraq, Sierra Leone and Timor-Leste. For many of these countries, the pattern included an initial decline, followed by a rapid rise—overshooting the initial trend, then a return to the pre-shock era (S3 Fig).

After excluding the regions of sub-Saharan Africa and Eastern Europe & Central Asia from the validation analyses, there was no significant difference between the 1950–2005 model predictions of country rates of improvement for life expectancy and the existing 2005–2009 rates (*t* = 0.52, p = 0.61 mean of the differences = 0.08 months per year (95% CI: -0.23; 0.40)). Plots of the standardized residuals and fitted values for rates of improvement of life expectancy showed no pattern.

There was also no statistically significant difference in the rate of improvements when the analysis was separated by gender (S1 Table) or when repeated for the period 1965–2010 (compared to 1950–2010) (S2 Table). In addition, taking the relative rate of improvement (Eq 9) also resulted in a deceleration scenario, where the global annual percentage change is declining by -0.015 (95% CI: -0.21, -0.09, p<0.001) (S3 Table).

Despite the above noted shocks due to conflict and the HIV epidemic, for all demographic indicators, the Bayesian Information Criterion (BIC) was lowest for the linear model (2) compared to the more complex quadratic variants (Eqs 7 & 8) (S4 Table). This suggests that there is no strong evidence for the use of a more complicated model and that a simple parametric model may be more descriptively accurate than the quadratic alternatives [34]. Furthermore, a linear model is also consistent with a mathematical approach to assess the presence of a deceleration or acceleration in the mortality indicators under study.

### Rate of child survival improvement

**Fig 3:**
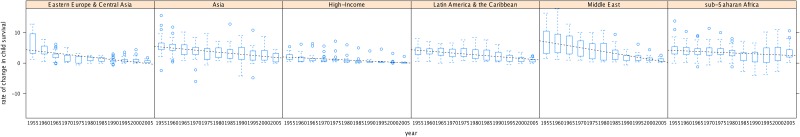
Regional changes in child survival (deaths per 1000 per year), 1950–2010. Note: mean regional trends in changes in child survival are indicated by the black dashed lines.

For child survival, the trends in improvements for the period 1950–2010 vary among regions (Fig 3). The global rate of improvement in child survival decreased from 4.4 to 1.6 deaths averted per 1000 per year, representing a deceleration of -0.063 deaths per year^2^ (95% CI: -0.081; -0.045, p < 0.001) for 1950–2010 (Table 1). Three of the six regions showed significant diminishing rates of improvements in child survival (e.g. Asia, Latin-America & the Caribbean and Eastern Europe & Central Asia) with two more marginally significant (e.g. sub-Saharan Africa, High-Income; p<0.10). The region with the lowest diminishing rates of improvement was High-income; it was also the region with the lowest rate of improvements in 1950–54. The most pronounced diminishing rates of improvements were observed in the Middle East, however; again it was the region with the highest variance. At the country level, the variance in *τ*_0,*c*_ and *τ*_*1*,*c*_ was -0.042 deaths per 1000 per year (p<0.05) and 0.001 deaths per 1000 per year^2^ (p<0.1) respectively.

### Rate of adult survival improvement

**Fig 4:**
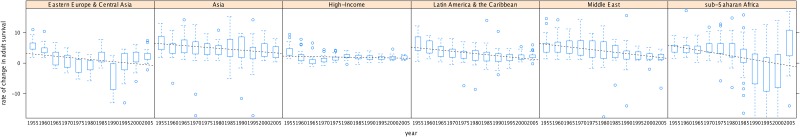
Regional changes in adult survival (deaths per 1000 per year), 1950–2010. Note: mean regional trends in changes in adult survival are indicated by the black dashed lines.

For adult survival for the period 1950–2010 (Fig 4), sub-Saharan Africa deviates from linear trends in the rate of improvements, with a shock peak in 1995–99, consistent with that observed in child survival and life expectancy. In addition, many former Soviet states (within the Eastern Europe & Central Asia region) demonstrated a shock around the 1980s. The global mean rate of improvement for adult survival declined from 4.8 to 3.7 deaths averted per 1000 adults per year for 1950–2010, representing statistically significant deceleration of -0.075 deaths per year^2^ (95% CI: -0.12; -0.05, p < 0.001). All six regions showed statistically significant diminishing improvements (Table 2). The High-income region was the region with the lowest initial rate of improvement in 1950–54 and the lowest deceleration over the study time period. At the country level, the variance in *τ*_0,*c*_ and *τ*_*1*,*c*_ was -0.052 deaths per 1000 per year (p<0.05) and 0.004 deaths per 1000 per year^2^ (p<0.001), respectively.

### Rate of senior survival improvement

**Fig 5:**
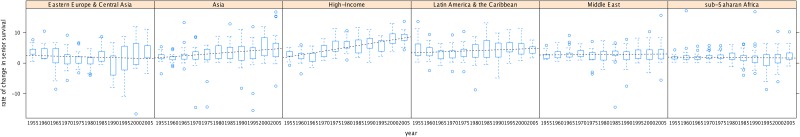
Regional changes in senior survival (deaths per 1000 per year), 1950–2010. Note: mean regional trends in changes in senior survival are indicated by the black dashed lines.

For senior survival for the period 1950–2010 (Fig 5), the global mean rate of improvement for senior survival increased from 2.4 to 4.2 deaths averted per 1000 seniors per year for 1950–2010, representing a potential acceleration of 0.031 deaths per year^2^ (95% CI: -0.01; 0.07, p < 0.15). No regions were found to be decelerating. Only Asia and High-income regions had statistically significant accelerations (p<0.05), with Latin America & the Caribbean being marginally significant (p<0.10). At the country level, the variance in *τ*_0,*c*_ and *τ*_*1*,*c*_ was -0.023 deaths per 1000 seniors per year (p<0.10) and 0.003 deaths per 1000 seniors per year^2^ (p<0.001), respectively. Although the global absolute rate of improvement increased over this time period, the relative rate of improvement declined from 2.6% per year to 1.7% per year. This discrepancy in relative versus absolute rates of improvements was largely due to the significant change in global 60p20 values, which almost tripled from 113 per 1000 seniors in 1950–54 to 314 per 1000 seniors in 2005–2010 (S3 Table).

## Discussion

**Fig 6:**
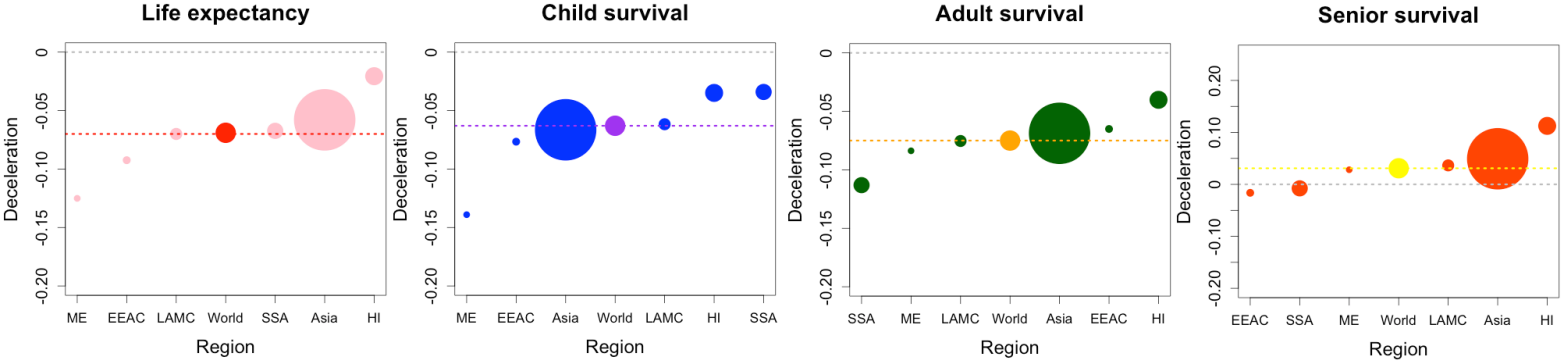
Mean regional trends in deceleration in life expectancy (months per year^2^), child survival, adult survival and senior survival (deaths per 1,000 per year^2^). Note: Size of marker is proportional to the region population size. Confidence intervals are indicated in Table 2. ME = Middle East; A = Asia; SSA = sub-Saharan Africa; LAMC = Latin American & the Caribbean; HI = High-income; EECA = Eastern Europe and Central Asia.

This paper quantifies the trends in the rate of life expectancy improvements at the global and regional levels over a large time period (1950–2010), in addition to the trends in the rates of child, adult and senior survival improvements (Fig 6), based on UN estimates.

Some previous studies [1,3,6,7] assumed a high degree of linearity of life expectancy (or child mortality) over time [21,22], discounting small diminishing rates of improvements which may add confusion to the interpretation of the trends. Interestingly, both White [6] and Lee [7] have found statistically significant fits with the levels of life expectancy and time using non-linear (polynomial) functions but opted for simpler regression approximations. Given continued improvements in life expectancy between 1950 and 2010 globally and the high degree of linearity over time, it is reasonable to overlook small declines in the rate of improvements. Indeed, linear approximations (such as Taylor Series) can be used under certain conditions to estimate trends in more complex, non-linear functions. The shift in perspective from the value of life expectancy to the rates of improvements in life expectancy can highlight small changes in deceleration previously considered as random errors in simpler linear models. For example, in its projections, the UN assumes slower gains at higher life expectancy, and takes into account both the relative improvements of a given country and the historical experience of other countries under similar conditions [29]. In this study, we use a simple linear mixed effects model of the rates of improvement to explicitly explore the concept of acceleration/deceleration at the global and regional levels; we also explicitly test higher power models (e.g. quadratic polynomials) but conclude the fitness is best with a simple linear model.

A rapid change in the rate of improvements at the region or country level graphically captures the effects of a change in health in that geographic area. This can provide a mechanism toward improved understanding of the determinants of health. Notably, diminishing rate of improvements followed by increasing rate of improvements can help highlight unusual events in a country trajectory. Some examples are: the initial impact of the AIDS epidemic and its subsequent response with the scale-up of antiretroviral therapy on high HIV prevalent countries (S2 Fig), premature adult mortality due to increased hazardous alcohol intake particularly in former Soviet states [35], China’s Great Famine of 1958–61 [36], the wars in Cambodia, Iraq, Sierra Leone, East Timor, and the ex-Yugoslavia (Bosnia and Herzegovina) [37].

Life expectancy improvements are complex to interpret. Indeed, as early-age mortality is reduced to lower levels, the potential gains in life expectancy are also diminished. Since mortality risk in midlife is lower, preventing a child death leads to more substantial life expectancy improvements than preventing an adult death. Any interpretation of accelerating/decelerating trends in life expectancy must keep in mind this historical perspective. In this respect, we investigated whether life expectancy diminishing rates of improvements arise from waning child survival gains achieved in the mid-twentieth century to fight infectious diseases or whether more recent innovations (e.g. related to cardiovascular treatment and reduced smoking) dissipate among the elderly. We found significant global deceleration in child survival over the study period; although variations existed among different regions. With 98% of children born surviving to age 5 in high-income countries [38], it is reasonable to expect little low-cost breakthrough innovations to substantially increase improvements in child survival. Nonetheless, all regions continue to have declines in child mortality.

For adult survival, the global deceleration in adult survival was equal in magnitude to that of child survival. Interestingly, adult survival was the only mortality indicator to be consistently decelerating across all regions. This deceleration may be due to the significant rise of non-communicable diseases (NCDs) in low- and middle-income countries [39] and diminishing returns in adult survival in the High-income region. Trends in innovative pharmaceutical compounds that could address NCDs have also been in decline over the last ten years [40]. Nonetheless, a global deceleration in adult survival implies that decelerations in life expectancy are not solely due to waning child survival improvements. Despite the deceleration effects in adult survival, absolute adult survival continues to rise and is likely to do so for many more decades.

The distribution of diminishing rates of adult survival improvements among regions and countries may reflect the variation in the rate at which age-standardized mortality from NCDs is falling in middle-income countries [41], and the impact of the increasing marginal cost of innovation to improve adult survival [38]. Countries and regions with constant or increasing rates of adult survival improvements may have the income and/or means to achieve higher rates of diffusion for health innovations, including medical technologies, health systems improvements or public health policies such as those devoted to tobacco cessation. Importantly, cost-effective health interventions to increase adult survival in low- and middle-income countries may not be fully implemented, notably tobacco control measures [8]. There may be concurrent and distinct improvements in both child and adult mortality across countries. Whereas lower income countries may be realizing their improvements primarily from reduced infectious disease mortality and to a lesser extent reduced NCD mortality; higher income countries may be realizing theirs via the inverse combination. This result may be interpreted to support the claim that there would not necessarily be diminishing returns at high life expectancy levels albeit via a change in the epidemiological causes of improvement [2].

For the High-income region, our analysis shows a potential steady growth scenario for overall life expectancy. This is consistent with our previous study [38], which showed that maximum life expectancy (approximating the mean life expectancy in higher income countries) continues to rise linearly over the last four decades. Given the decelerations in child and adult improvements, the steady growth in life expectancy improvements is likely due to a combination of lower magnitudes of deceleration in child and adult improvement (compared to the other regions) and to the significant acceleration in senior survival improvements. Indeed, the High-income region is comprised of countries previously classified as the best-performing countries [1,3]. While other studies [1,3,6,7] have previously shown linear growth for life expectancy among High-income countries, this continues to be astounding.

Our analysis presents limitations. First, we did not consider the trends in very old-age mortality. Certainly, Christensen and colleagues [42] show that more recent increases in life expectancy in high-income countries have been a result of declines in mortality at the ages of 80 years and older, which leads to an almost linear increase in life expectancy as described by White [6]. Our study, however, was limited by available data, and global mortality rates for above 80 years old for low-, middle- and high-income countries alike were not presently available for investigation. Second, we used UN mortality estimates [28,43] which rely on all data available including censuses, surveys, vital registers, international databases and specific modeling assumptions [44]. For higher income countries, very good sources of data exist (e.g. Human Mortality Database [45]) which are directly incorporated into the UN estimates. Yet, for countries with limited data, UN estimates are extrapolated with model life tables [44]. We used UN estimates in order to have a consistent set of numbers—that have been peer-reviewed—for all the countries analyzed. However, we acknowledge that given the lack of quality data for a number of countries including the absence of surveys and censuses for the earlier years (e.g. 1950s, 1960s), there is substantial uncertainty in estimating historical health indicators for a number of low- and middle-income countries; data sources (summary, type of data and reference date) and the methods used to derive each country’s UN mortality estimates are available on their World Population Prospects (2012 Revision) website [46]. In this respect, we performed additional analyses for all indicators from 1965–2010 (S2 Table) and noted no statistically significant difference in the trends in the rate of improvements. Third, even within regions, substantial heterogeneity exists across countries, as countries have not reached the same stage of epidemiological transition, or as sub-regions have been impacted differently, for example by the HIV epidemic (e.g. Western Africa as opposed to Eastern and Southern Africa). Hence, our regional findings may be interpreted with caution and we provide country-specific results in the supplementary data (S1 Fig). In addition, we used a linear mixed effect model implying linear rates of improvements and only tested a limited number of higher order alternative models as we intended to quantify metrics of acceleration/deceleration. Yet, in many regions and countries, we identified specific times of rapid changes (e.g. decelerations followed by accelerations) (see S2 and S3 Figs) also examined elsewhere [47,48], which a linear model at a one-region or country-specific level may be inappropriate for characterizing. Finally, this analysis did not examine the specific causes of some of the findings, which we left for future work with the use of appropriate statistical analysis and covariates/determinants (e.g. income, education). Specifically, the main objective of the paper is to present a model to estimate trends in the rate of improvement of mortality indicators. In doing so, we estimated acceleration or deceleration on average during specific periods without describing the underlying causes or circumstances. Rather, our approach can point to the regions/countries and time periods where unusual events occurred (S2 and S3 Figs), which may call for subsequent examination and incorporation into the models used including for example a perturbation term (e.g. HIV prevalence term) to account for the potential shocks encountered. That being said, the presence of these temporary shocks may alter the intuitive appeal of steady diminishing/increasing returns in mortality declines.

In this paper, using UN data, we computed explicitly a mathematical definition of deceleration or declining rates of life expectancy improvements. We found that the global rate of improvements in life expectancy has been decreasing over time. Furthermore, we also found that the improvements for child and adult survival were rising at a slower pace however senior survival was rising at an accelerated pace. Given the progress toward MDG 4 [21], the epidemiologic transition may well take place through diminishing improvements in under-five mortality, steady improvements in adult mortality and then to improvements above age 60. Like Vallin and Meslé [1] and Christensen et al. [42], we do not rule out further rises in life expectancy past a hypothetical biological ceiling but point to potential gains in life expectancy at higher ages of life. Despite the public health issues that accompany the increase in adult population worldwide, there has been much less focus on adults. Nonetheless, variability in adult survival indicates current innovations may be sufficient to achieve a higher degree of acceleration. Further global efforts devoted to adult health focused on research and development for new tools and their widespread application, analogous to those implemented for child health, will enhance the accessibility of these innovations and reverse the slowing of adult survival improvements [49].

## Supporting Information

S1 Text (1) List of countries and (2) regions retained in the analysis. (DOCX)

S1 Fig Trends in improvements in life expectancy (months per year), all countries, 1950–2010. (TIF)

S2 Fig Trends in improvements in life expectancy (months per year) for select countries with high HIV-prevalence, 1950–2010. (TIF)

S3 Fig Trends in improvements in life expectancy (months per year) among select countries with natural disasters and conflicts, 1950–2010. (TIF)

S1 Table Results of the linear mixed effect model for the relationship between rate of improvements in life expectancy for male- and female-separated and combined from 1950–2010. (DOCX)

S2 Table Results of the linear mixed effect model for the relationship between rate of improvements in life expectancy, child, adult and senior survival from 1965–2010. (DOCX)

S3 Table Results of the linear mixed effect model for the relationship between relative rate of improvements in life expectancy, child, adult and senior survival from 1950–2010. (DOCX)

S4 Table Bayesian Information Criterion (BIC) for selection of linear and quadratic mixed effect models. (DOCX)
