# Supplementary material for: Are Global and Regional Improvements in Life Expectancy and in Child, Adult and Senior Survival Slowing?
Source: PLoS One. 2015 May 18;10(5):e0124479. doi: 10.1371/journal.pone.0124479 (PMC4436293; doi:10.1371/journal.pone.0124479)
Supplement: S1 Text — (DOCX) [file pone.0124479.s001.docx]

**Text S1 – (1) List of countries and (2) regions retained in the analysis.**

**1. List of countries retained in the analysis.**

Afghanistan Albania Algeria Angola Argentina, Armenia Australia Austria Azerbaijan, Bahrain, Bangladesh, Belarus, Belgium, Benin, Bhutan Bolivia (Plurinational State of), Bosnia and Herzegovina, Botswana, Brazil, Bulgaria, Burkina Faso, Burundi, Cambodia, Cameroon, Canada, Central African Republic, Chad, Chile, China, China - Hong Kong SAR, Colombia, Comoros, Congo, Costa Rica, Côte d'Ivoire, Croatia, Cuba, Cyprus, Czech Republic, Dem. People's Republic of Korea, Democratic Republic of the Congo, Denmark, Djibouti, Dominican Republic, Ecuador, Egypt, El Salvador, Equatorial Guinea, Eritrea, Estonia, Ethiopia, Fiji, Finland, France, Gabon, Gambia, Georgia, Germany, Ghana, Greece, Guatemala, Guinea, Guinea-Bissau, Guyana, Haiti, Honduras, Hungary, India, Indonesia, Iran (Islamic Republic of), Iraq, Ireland, Israel, Italy, Jamaica, Japan, Jordan, Kazakhstan, Kenya, Kuwait, Kyrgyzstan, Lao People's Democratic Republic, Latvia, Lebanon, Lesotho, Liberia, Libya, Lithuania, Madagascar, Malawi, Malaysia, Mali, Mauritania, Mauritius, Mexico, Mongolia, Montenegro, Morocco, Mozambique, Myanmar, Namibia, Nepal, Netherlands, New Zealand, Nicaragua, Niger, Nigeria, Norway, Oman, Pakistan, Panama, Papua New Guinea, Paraguay, Peru, Philippines, Poland, Portugal, Puerto Rico, Qatar, Republic of Korea, Republic of Moldova, Reunion, Romania, Russian Federation, Rwanda, Saudi Arabia, Senegal, Serbia, Sierra Leone, Singapore, Slovakia, Slovenia, Somalia, South Africa, South Sudan, Spain, Sri Lanka, State of Palestine, Suda, Swaziland, Sweden, Switzerland, Syrian Arab Republic, Tajikistan, TFYR Macedonia, Thailand, Timor-Leste, Togo, Trinidad and Tobago, Tunisia, Turkey, Turkmenistan, Uganda, Ukraine, United Arab Emirates, United Kingdom, United Republic of Tanzania, United States of America, Uruguay, Uzbekistan, Venezuela (Bolivarian Republic of), Viet Nam, Yemen, Zambia, Zimbabwe.

**2. List of regions retained in the analysis.**

**Asia**

This region includes the following countries: Afghanistan, Bangladesh, Bhutan, Cambodia, China, China - Hong Kong SAR, Dem. People's Republic of Korea, Fiji, India, Indonesia, Iran (Islamic Republic of), Lao People's Democratic Republic, Malaysia, Mongolia, Myanmar, Nepal, Pakistan, Papua New Guinea, Philippines, Republic of Korea, Singapore, Sri Lanka, Thailand, Timor-Leste, Viet Nam. This region is composed of countries within the United Nations (UN) sub-regions of Southern Asia, South-Eastern Asia, Eastern Asia and Melanesia, excluding those identified in the High-income region.

**Eastern Europe & Central Asia**

This region includes the following countries: Albania, Belarus, Bosnia and Herzegovina, Bulgaria, Croatia, Czech Republic, Estonia, Hungary, Kazakhstan, Kyrgyzstan, Latvia, Lithuania, Montenegro, Poland, Republic of Moldova, Romania, Russian Federation, Serbia, Slovakia, Slovenia, Tajikistan, TFYR Macedonia, Turkmenistan, Ukraine, Uzbekistan. This region is composed of countries within the UN sub-regions of Eastern Europe, Central Asia, Northern Europe and Southern Europe, excluding those identified in the High-income region.

**High-income**

This region includes the following countries: Australia, Austria, Belgium, Canada, Denmark, Finland, France, Germany, Greece, Ireland, Italy, Japan, Netherlands, New Zealand, Norway, Portugal, Spain, Sweden, Switzerland, Turkey, United Kingdom, United States of America. This region is composed of the member countries from the Organization for Economic Co-operation and Development prior to 1980.

**Latin America and the Caribbean**

This region includes the following countries: Argentina, Bolivia (Plurinational State of), Brazil, Chile, Colombia, Costa Rica, Cuba, Dominican Republic, Ecuador, El Salvador, Guatemala, Guyana, Haiti, Honduras, Jamaica, Mexico, Nicaragua, Panama, Paraguay, Peru, Puerto Rico, Trinidad and Tobago, Uruguay, Venezuela (Bolivarian Republic of). This region is composed of countries within the UN sub-regions of South America, Central America and the Caribbean, excluding those identified in the High-income region.

**Middle East**

This region includes the following countries: Algeria, Armenia, Azerbaijan, Bahrain, Cyprus, Egypt, Georgia, Iraq, Israel, Jordan, Kuwait, Lebanon, Libya, Morocco, Oman, Qatar, Saudi Arabia, State of Palestine, Sudan, Syrian Arab Republic, Tunisia, United Arab Emirates, Yemen. This region is composed of countries within the UN sub-regions of Northern Africa and Western Asia, excluding those identified in the High-income region.

**Sub-Saharan Africa**

This region includes the following countries: Angola, Benin, Botswana, Burkina Faso, Burundi, Cameroon, Central African Republic, Côte d'Ivoire, Chad, Comoros, Congo, Democratic Republic of the Congo, Djibouti, Equatorial Guinea, Eritrea, Ethiopia, Gabon, Gambia, Ghana, Guinea, Guinea-Bissau, Kenya, Lesotho, Liberia, Madagascar, Malawi, Mali, Mauritania, Mauritius, Mozambique, Namibia, Niger, Nigeria, Reunion, Rwanda, Senegal, Sierra Leone, Somalia, South Africa, South Sudan, Swaziland, Togo, Uganda, United Republic of Tanzania, Zambia, Zimbabwe. This region is composed of countries within the UN sub-regions of Eastern Africa, Western Africa, Middle Africa and Southern Africa.

Note: the original data sources for the derived UN estimates can be found in the meta info file link:

http://esa.un.org/unpd/wpp/Excel-Data/WPP2012_F02_METAINFO.xls
