# Supplementary figures and images for: Are Global and Regional Improvements in Life Expectancy and in Child, Adult and Senior Survival Slowing?
Source: PLoS One. 2015 May 18;10(5):e0124479. doi: 10.1371/journal.pone.0124479 (PMC4436293; doi:10.1371/journal.pone.0124479)

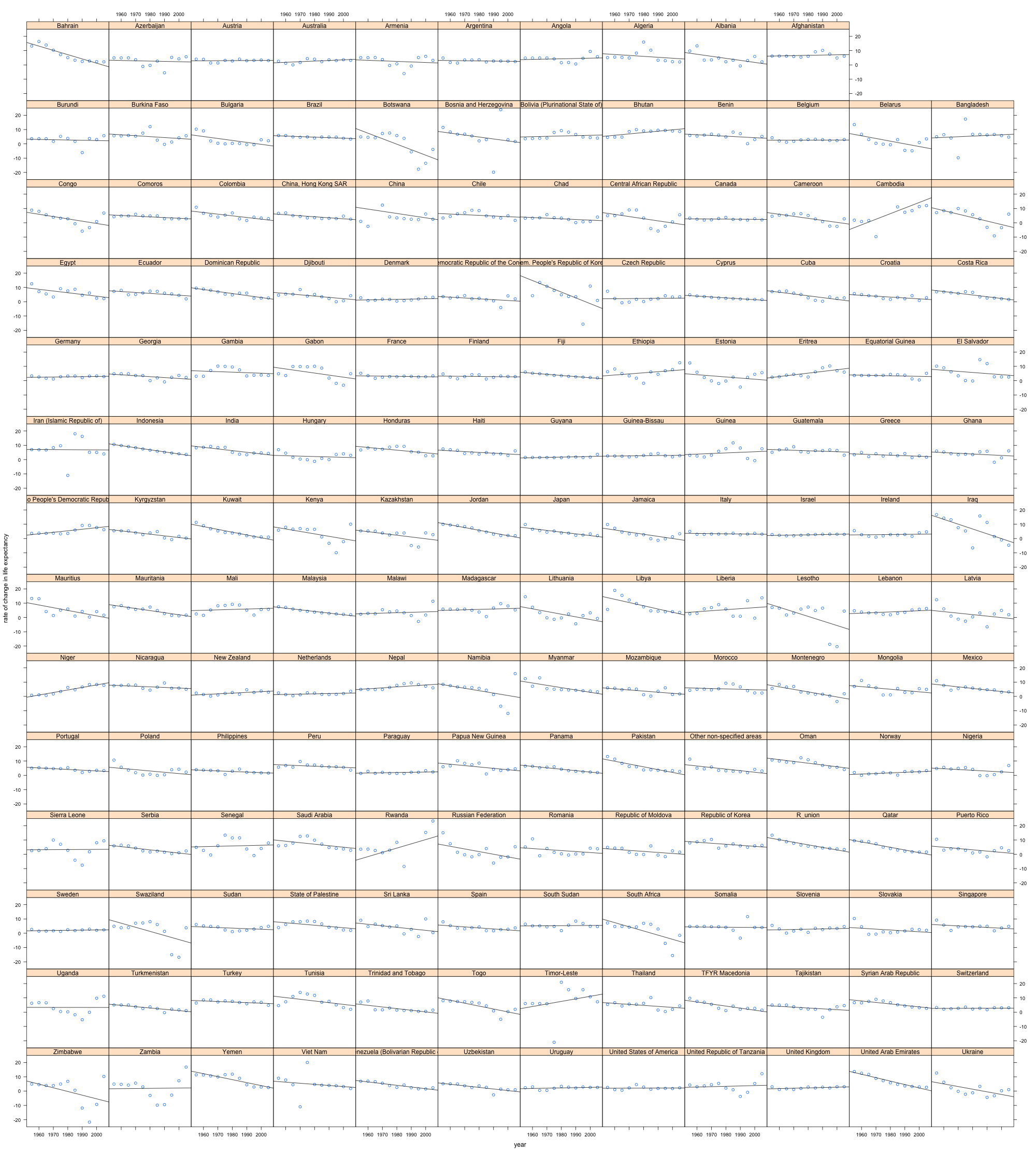

Supplement: S1 Fig — (TIF) [file pone.0124479.s002.tif]

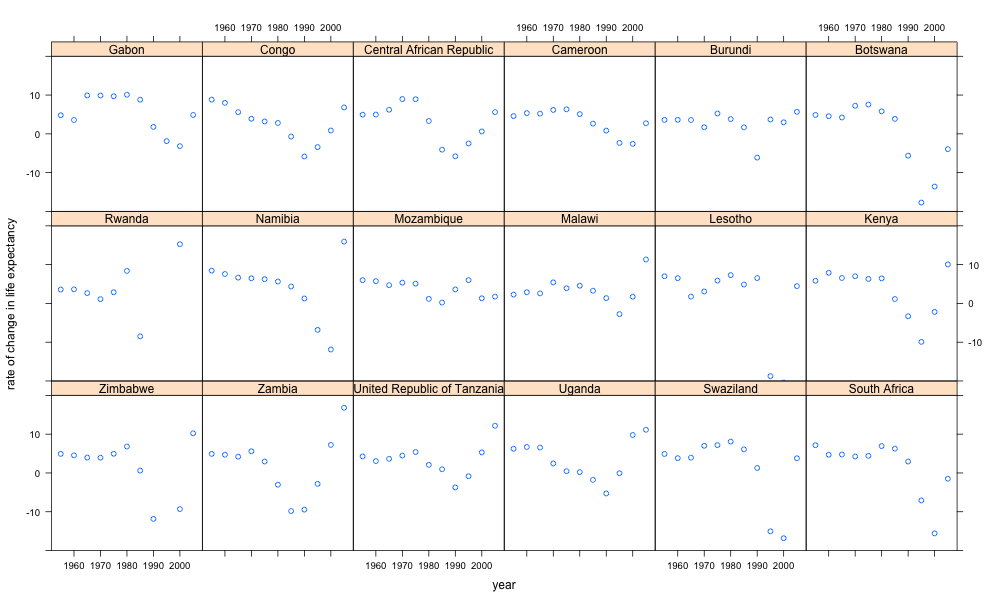

Supplement: S2 Fig — (TIF) [file pone.0124479.s003.tif]

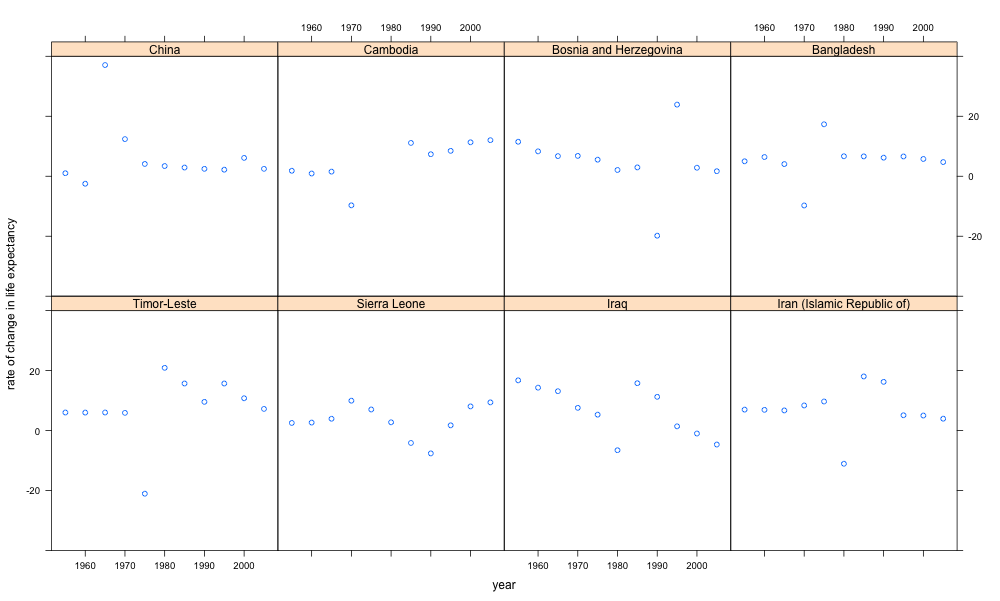

Supplement: S3 Fig — (TIF) [file pone.0124479.s004.tif]
