# Supplementary material for: Are Global and Regional Improvements in Life Expectancy and in Child, Adult and Senior Survival Slowing?
Source: PLoS One. 2015 May 18;10(5):e0124479. doi: 10.1371/journal.pone.0124479 (PMC4436293; doi:10.1371/journal.pone.0124479)
Supplement: S1 Table — (DOCX) [file pone.0124479.s005.docx]

**Text S5. Results of the linear mixed effect model for the relationship between rate of improvements in life expectancy for male- and female-separated and combined from 1950-2010.**

|  | **Life Expectancy**  **(male)** | **Life Expectancy**  **(female)** | **Life Expectancy**  **(both)** |
| --- | --- | --- | --- |
| Marginal R^2^ | 0.032 | 0.069 | 0.05 |
| Conditional R^2^ | 0.15 | 0.22 | 0.17 |
| Intercept  (Rate of improvement at beginning of study period) | 5.4***  (0.49) | 6.2***  (0.43) | 5.8***  (0.46) |
| Time  (Trend in rate of improvement) | -0.061***  (0.015) | -0.084***  (0.014) | -0.073***  (0.015) |
| Mean rate of improvement at end of study period | 4.0  (0.25) | 4.0  (0.27) | 4.0  (0.26) |

*** indicates p < 0.001.

S.E. (standard error) is in parentheses. The intercept is in units of months per year or deaths per 1000 per year. The time effects are in units of months per year^2^ or deaths per 1000 per year^2^.
