# Supplementary material for: Are Global and Regional Improvements in Life Expectancy and in Child, Adult and Senior Survival Slowing?
Source: PLoS One. 2015 May 18;10(5):e0124479. doi: 10.1371/journal.pone.0124479 (PMC4436293; doi:10.1371/journal.pone.0124479)
Supplement: S2 Table — (DOCX) [file pone.0124479.s006.docx]

**Text S6. Results of the linear mixed effect model for the relationship between rate of improvements in life expectancy, child, adult and senior survival from 1965-2010.**

|  | **Life Expectancy** | **Child Survival** | **Adult Survival** | **Senior**  **Survival** |
| --- | --- | --- | --- | --- |
| Marginal R^2^ | 0.02 | 0.031 | 0.018 | 0.025 |
| Conditional R^2^ | 0.18 | 0.17 | 0.15 | 0.10 |
| Intercept  (Rate of improvement at beginning of study period) | 4.7***  (0.56) | 3.8***  (0.38) | 3.6**  (0.61) | 2.4***  (0.18) |
| Time  (Trend in rate of improvement) | -0.060**  (0.022) | -0.058***  (0.010) | -0.070**  (0.033) | 0.040*  (0.017) |
| Mean rate of improvement at end of study period | 4.0  (0.25) | 1.6  (0.14) | 3.7  (0.40) | 4.2  (0.28) |

* indicates p < 0.05, ** indicates p < 0.01; *** indicates p < 0.001.

S.E. (standard error) is in parentheses. The intercept is in units of months per year or deaths per 1000 per year. The time effects are in units of months per year^2^ or deaths per 1000 per year^2^.
