# Supplementary material for: Are Global and Regional Improvements in Life Expectancy and in Child, Adult and Senior Survival Slowing?
Source: PLoS One. 2015 May 18;10(5):e0124479. doi: 10.1371/journal.pone.0124479 (PMC4436293; doi:10.1371/journal.pone.0124479)
Supplement: S3 Table — (DOCX) [file pone.0124479.s007.docx]

**Text S7. Results of the linear mixed effect model for the relationship between relative rate of improvements in life expectancy, child, adult and senior survival from 1950-2010.**

|  | **Life Expectancy** | **Child Survival** | **Adult Survival** | **Senior**  **Survival** |
| --- | --- | --- | --- | --- |
| Intercept  (% improvement at beginning of study period) | 1.02***  (0.16) | 0.54***  (0.052) | 0.82***  (0.10) | 2.6***  (0.30) |
| Time  (Trend in rate of improvement) | -0.015***  (0.003) | -0.0091***  (0.002) | -0.014***  (0.003) | -0.024***  (0.007) |
| Mean rate of improvement at end of study period (%) | 0.55  (0.045) | 0.18  (0.016) | 0.59  (0.09) | 1.73  (0.12) |

‘ indicates p ~ 0.15, * indicates p < 0.05, ** indicates p < 0.01; *** indicates p < 0.001.
