# Supplementary material for: Are Global and Regional Improvements in Life Expectancy and in Child, Adult and Senior Survival Slowing?
Source: PLoS One. 2015 May 18;10(5):e0124479. doi: 10.1371/journal.pone.0124479 (PMC4436293; doi:10.1371/journal.pone.0124479)
Supplement: S4 Table — (DOCX) [file pone.0124479.s008.docx]

**Text S8. Bayesian Information Criterion (BIC) for selection of linear and quadratic mixed effect models.**

|  | Change in demographic indicator ($\nu$) | | | | |
| --- | --- | --- | --- | --- | --- |
| Model | e_o,m_ | e_o,f_ | 5q0 | 45q15 | 20q60 |
| Linear  $v_{i,Y}=\gamma_{0}+\gamma_{1}t$ | 10222 | 9946 | 8925 | 10825 | 8697 |
| Quadratic 1  $v_{i,Y}=\gamma_{0}+\gamma_{1}t^{2}$ | 10238 | 9969 | 8998 | 10847 | 8749 |
| Quadratic 2  $v_{i,Y}=\gamma_{0}+\gamma_{1}t^{2}+\gamma_{1}t$ | 10261 | 9981 | 9032 | 10849 | 8764 |

Note: For all demographic indicators, the BIC was lowest for the linear model. For the comparison between the linear and quadratic 1 models, *t*=-3.4, difference = -37.2 (-67.5; -6.9), p<0.05. For the comparison between the linear and quadratic 2 models, t=-3.6, difference = -54.4 (=95.9; -12.9), p<0.05.
